# Care of Terminally ill Cancer Patients: An Intensivist’s Dilemma

**DOI:** 10.4103/0973-1075.73671

**Published:** 2010

**Authors:** Sukhminder Jit Singh Bajwa, Sukhwinder Kaur Bajwa, Jasbir Kaur

**Affiliations:** Department of Anaesthesiology and Intensive Care, Gian Sagar Medical College and Hospital, Ram Nagar, Banur, Punjab, India

Sir,

With respect to the letter in response to our article I am giving the reply to the concerned query. I will be sending the same to the person concerned also.

Resource allocation is definitely a major problem especially in government institutes of our country where one can hardly find any bed or ventilators available in the intensive care unit (ICU) for critically sick patients. Economic considerations are the major factor, which accounts for the 100% bed occupancy in these ICUs in a developing country like ours.

Although our institute may be a private one, it is however being run by a charitable trust. As per the management policy, we have committed ourselves in the free treatment of at least 30% of the total patients. Criteria for such treatment include proven economic and social status of the concerned patient, which is being very well managed by the marketing department of the institute. The main ICU is 12 bedded, which usually has bed occupancy of 80–90% over the year. We have a step-down ICU, which is 3 bedded, fully equipped with both ventilators and monitors.

The incidence which prompted us to undertake this retrospective study was non-availability of beds for two young patients of polytrauma who required ICU admission. At that time all the beds were occupied and these young patients were denied admission. Thereafter, we modified our admission criteria and priorities were set again. We always try to keep at least one bed free in our main ICU and utilize the services of the step-down ICU.

With the help from hospital management committee, we have been able to maintain a good balance between the resources availability and the demand for the same. The corresponding author is himself an MBA degree holder, besides being a specialist in the intensive care; this has helped us in managing the allocation of resources to a good extent. As a matter of fact, all the decisions regarding the admissions of patients to ICU are completely taken by us and this eases our task in setting priorities and resource allocation. In addition to that we have never faced any problem of funding of poor and needy patients and the hospital management committee stands right behind us during these circumstances.

The social and moral grounds for resource allocation are very well explained by the article itself. Rather, our ICU has gained a lot of popularity in the surrounding areas and we just want to convey that the institute is just four years old. Thanks for writing to us and any other queries are most welcome.

